# Seasonal variations in onset and exacerbation of inflammatory bowel diseases in children

**DOI:** 10.1186/s13104-015-1702-y

**Published:** 2015-11-20

**Authors:** Rajmohan Dharmaraj, Anas Jaber, Rajan Arora, Karen Hagglund, Hernando Lyons

**Affiliations:** Department of Pediatrics, St. John Providence Children’s Hospital, Detroit, MI 48236 USA; Department of Biostatistics, St. John Providence Children’s Hospital, Detroit, MI 48236 USA; Department of Pediatric Gastroenterology, St. John Providence Children’s Hospital, Wayne State University School of Medicine, Detroit, MI 48236 USA

## Abstract

**Background:**

Studies have suggested that inflammatory bowel diseases (IBD) follow a seasonal pattern with regard to their onset and exacerbations. The aim of this study is to determine if there is any seasonal pattern to the onset and exacerbation of IBD in the pediatric population and if the birth of children diagnosed with IBD follows a seasonal pattern.

**Methods:**

Patients between the ages of 1 and 21 years and with a diagnosis of IBD established between July 1992 and July 2012 were included. Their onset and exacerbations of IBD (year and season) were recorded. The birth dates of the patients were aggregated to determine whether a seasonal birth pattern existed amongst them.

**Results:**

A total of 170 children were included in this study; 34 % of patients had their onset in the fall and 19 % of them had their onset in the summer. The total number of documented exacerbations was 358 and the median number of exacerbations was two, with a range of 1–11. IBD exacerbations were generally uniformly distributed throughout the year. We did not observe any specific season where children with IBD tended to be born.

**Conclusions:**

Our data suggests that the onset of symptoms of IBD tends to have a seasonal trend with the highest incidence in the fall. However, we did not observe any association between seasonality and exacerbations in the pediatric population. Moreover, there was no specific season in which children with IBD tended to be born in greater numbers.

## Background

Inflammatory bowel diseases (IBD) are chronic inflammatory conditions of the gastrointestinal tract with unknown etiology, characterized by exacerbations and remissions with no known triggering factors for either onset or exacerbation. The two major forms of the IBD are Crohn’s disease (CD) and ulcerative colitis (UC). A patient is classified as having indeterminate colitis (IC) if, after careful investigation, the differential diagnosis between CD and UC remains uncertain. UC and IC affect the colon whereas CD can involve any part of the gastrointestinal tract from the oral cavity to the anus [1].

Although the cause of IBD has not yet been determined, genetic and environmental factors are thought to play an important role in its etiology [2]. Existing data suggest that there is a dysfunctional mucosal immune response to bacteria in the pathogenesis of IBD, especially in the cause of CD [3]. Chronic inflammatory response may be triggered by bacterial or viral pathogens and/or due to a defective mucosal barrier. The search for causative or modulating risk factors in the environmental field is ongoing and some of them may be connected with public and personal hygiene. The risk of infections might also be subjected to climate, changes of biorhythm or other environmental events. This could potentially result in seasonal variations in the natural history of IBD [4].

Few studies have investigated the seasonal variations associated with the onset of IBD symptoms and the results are conflicting. Acute onset of symptoms has been reported more frequently in January and July with CD, and UC symptoms have been reported more frequently in December [5]. Data from an Italian study indicates that the onset of CD symptoms occurred more frequently during spring and summer season; a similar trend was observed with UC [6]. A prospective study published in Norway demonstrated that symptomatic onset of UC occurred more frequently in December and January; no seasonality was observed with CD [7]. However, other studies have reported no seasonal variations in the onset of symptoms in UC [8].

The relationship between seasonality and exacerbations of IBD requires further study, as the results to date have been inconsistent. The results of some studies suggest a degree of seasonality; increased rates of UC exacerbations were found in the spring and autumn for Swedish populations, while another study found highest exacerbations of CD in the autumn and winter for Canadian populations [9, 10]. Seasonality of UC has also been reported in few other studies [11–14]. However, some studies have not found any seasonal pattern of onset or exacerbations amongst patients with UC for British and American populations [15, 16]. Hospital admissions were reported more often in winter but no seasonality has been reported for emergency admissions [17].

Conflicting data has also been reported for the seasonal variation in births of patients with IBD. One study demonstrated that birth during the winter period in Israel was associated with an increased risk of developing CD whereas birth during the spring was associated with a reduced risk. Another study did not find any seasonality with respect to the month of birth for British populations less than 20 years of age [18, 19].

There has been little data reported on seasonal variations in the onset, exacerbation and birth cases of IBD patients within the pediatric population. Therefore, the aim of this study is to determine if there is any seasonal pattern with respect to the onset and exacerbations of IBD in the pediatric population and if the birth of children diagnosed with IBD follows a seasonal pattern.

## Methods

### Study design

This study was performed in the Pediatric Gastroenterology Clinic at St. John Providence Children’s Hospital, a tertiary referral center for the southeast Michigan community. Upon approval by the St. John Providence Children’s Hospital Institutional Review Board, patients aged 1–21 years with a diagnosis of IBD established between July 1992 and July 2012 were investigated concerning the onset of symptoms, exacerbations and their season of birth. The seasons were defined as winter (December–February), spring (March–May), summer (June–August) and fall (September–November). Data were collected by identifying and reviewing hospital charts and records of patients with established diagnosis of IBD using International Classification of Diseases (ICD) codes. In each case the diagnosis of IBD had been established by clinical, endoscopic, histological, and/or radiological criteria. The date of diagnosis was established according to the date or month of onset of symptoms where such a diagnosis of IBD could be defined. The effect of recall bias was reduced using the calendar season and by excluding the patients who claimed their symptoms began more than 6 months prior to their visit to our clinic. Exacerbation was defined as the recurrence or worsening of symptoms in the absence of secondary causes, which could only be controlled with a change or increase in the regularly used medication for the management of IBD. The date of exacerbation was obtained by reviewing records from outpatient visits, hospitalizations, emergency department visits and telephone encounters. Month of birth recorded in the hospital database was used to determine the season of birth.

### Statistical analysis

To assess the seasonality of IBD onset, exacerbation and birth cases within our study cohort, we pooled the data from all IBD patients to evaluate its general distribution with respect to the season. In addition, we also compared the individual data for the first, second and third exacerbations to assess any seasonal pattern. Data were analyzed using the non-parametric binomial test to assess the seasonality of IBD onset and birth cases, and Chi square test for IBD exacerbations. Statistical significance was set at a *p* value of 0.05. All calculations were performed using statistical package for social science (SPSS) version 14 (SPSS Inc, Chicago, IL, USA).

## Results

A total of 170 IBD patients with 358 exacerbations were investigated. Of the patients, 58 % were male, 78 % were Caucasians, 74 % were outpatients, and 52.9 % had CD. The mean age at the onset of symptoms was 13.3 ± 4.6 years [mean ± standard deviation (SD)], and the mean age for first exacerbation was 14.3 ± 3.9 years (mean ± SD).

**Fig. 1:**
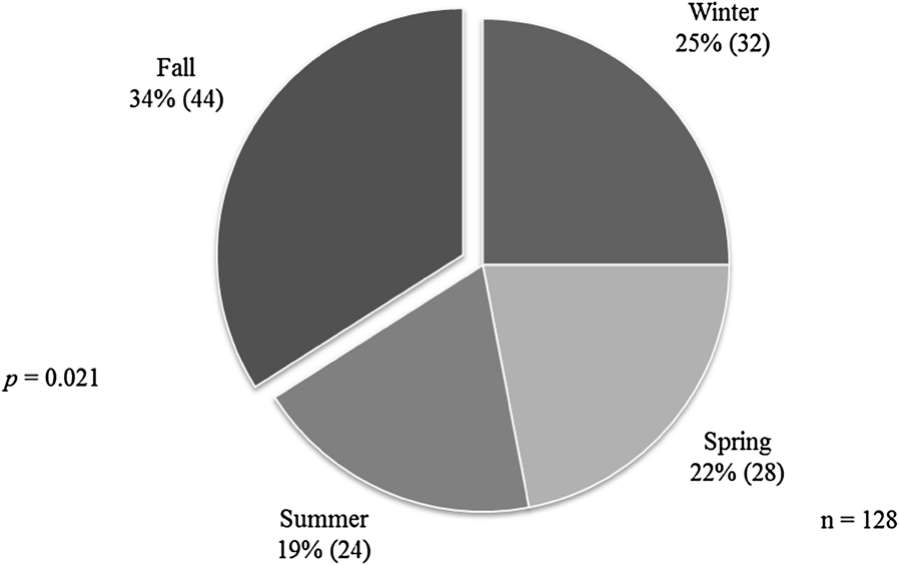
Pie chart depicting the distribution of onset of symptoms according to the season presented as percentage (n) in 128 patients with IBD. A significantly larger proportion of patients experienced their symptoms during the fall than during other seasons (*p* = 0.021). *IBD* inflammatory bowel diseases

**Fig. 2:**
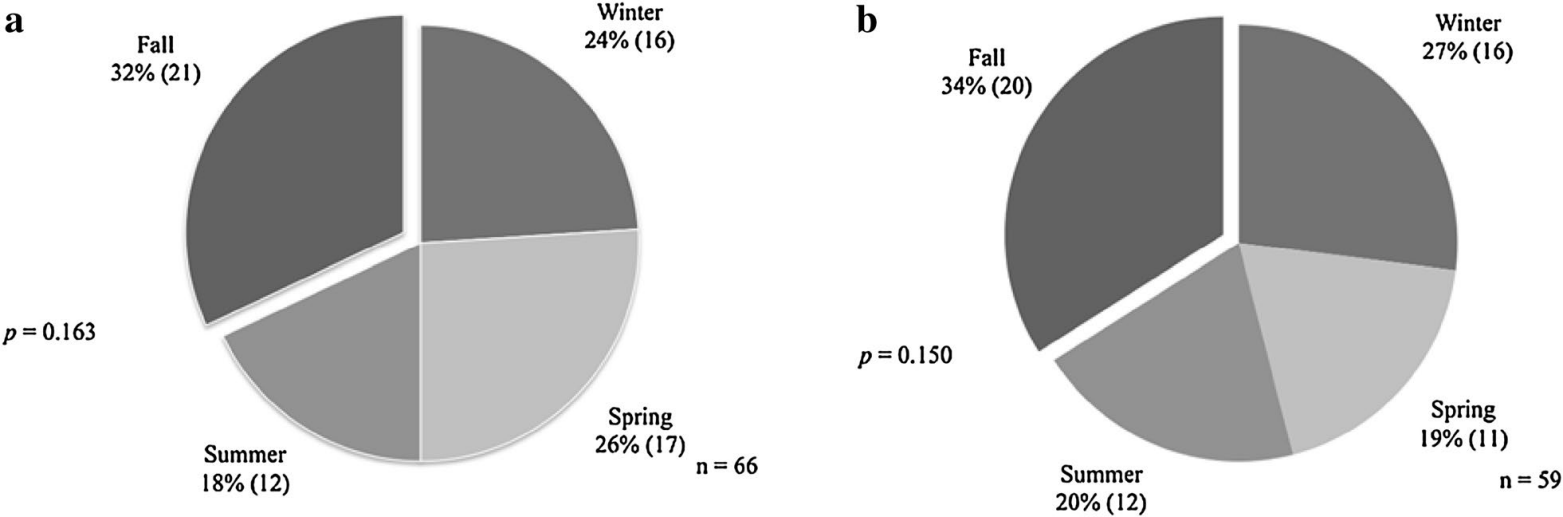
Distribution of onset of symptoms according to the season. Distribution presented as percentage (n) in 66 patients with UC. The onset of symptoms were noted to be more frequently in the fall (32 %) than during other seasons but with no statistical significance (*p* = 0.163) (**a**). Similar findings were observed in 59 patients with CD; a larger proportion of patients experienced their symptoms during the fall (34 %) than during other seasons but with no statistical significance (*p* = 0.150) (**b**). *UC* ulcerative colitis, *CD* Crohn’s disease

**Fig. 3:**
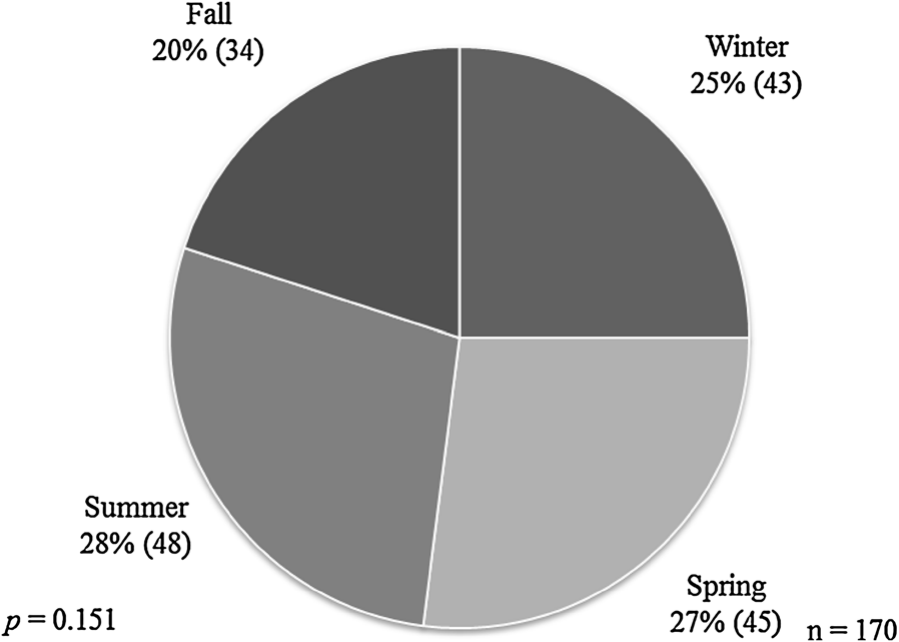
Pie chart showing the seasonal distribution of birth cases among 170 patients with IBD expressed as percentage (n). All four seasons had roughly similar percentage of births with no statistical significance (*p* = 0.151). *IBD* inflammatory bowel diseases

Forty-two patients were excluded from the analysis of onset; 21 of these patients had incomplete records, 18 were not able to give a precise date of the onset of their symptoms and three patients had IC. The distribution of onset of symptoms according to the season is shown in Table 1 and Fig. 1. By mere chance we expected that the onset of symptoms should occur equally in the four seasons. However, we noticed that 34 % of patients with IBD experienced their onset in the fall and 19 % in the summer. The rest were split somewhat equally between the winter and spring, with the results being statistically significant. When breaking down the study group to UC and CD categories, the onset of the diseases continued to be more frequently in fall but with no statistical significance (Table 1; Fig. 2). Birth dates were aggregated, and the four seasons had roughly similar percentage of births with no statistical significance (*p* = 0.151) (Fig. 3).

**Table 1:** Distribution of onset of symptoms according to season

Season	IBD (n = 128)	UC (n = 66)	CD (n = 59)
Winter, n (%)	32 (25)	16 (24)	16 (27)
Spring, n (%)	28 (22)	17 (26)	11 (19)
Summer, n (%)	24 (19)	12 (18)	12 (20)
Fall, n (%)	44 (34)	21 (32)	20 (34)
*p*	0.021	0.163	0.150

*IBD* inflammatory bowel diseases, *UC* ulcerative colitis, *CD* Crohn’s disease

**Fig. 4:**
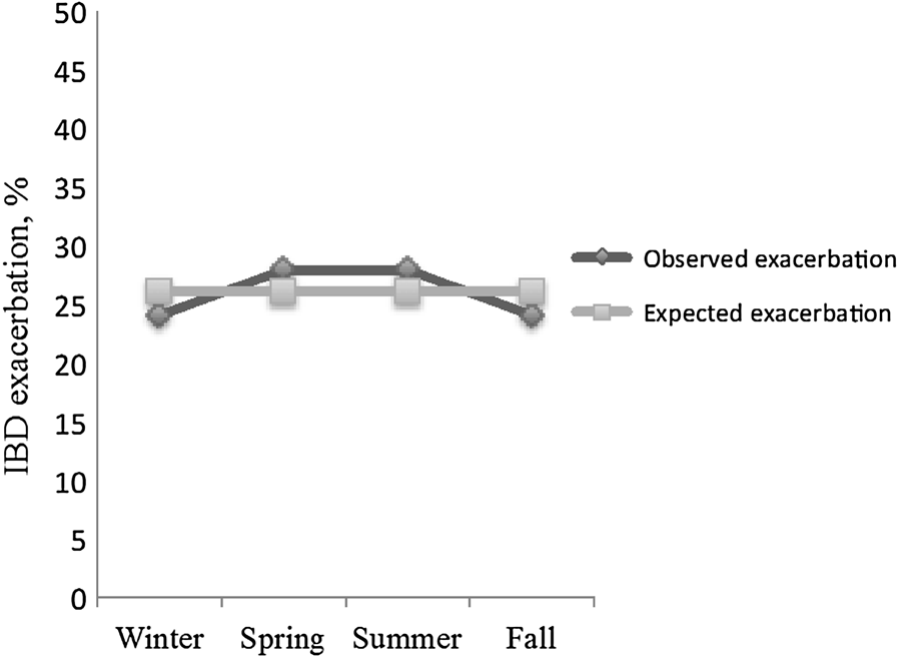
Seasonal distribution of the IBD exacerbations (n = 358) among 170 patients as compared to expected pattern of exacerbations expressed as percentage in a 3-D line graph. No difference was detected in seasonality between the observed and expected pattern of IBD exacerbations experienced by patients (*p* = 0.19). *IBD* inflammatory bowel diseases

**Fig. 5:**
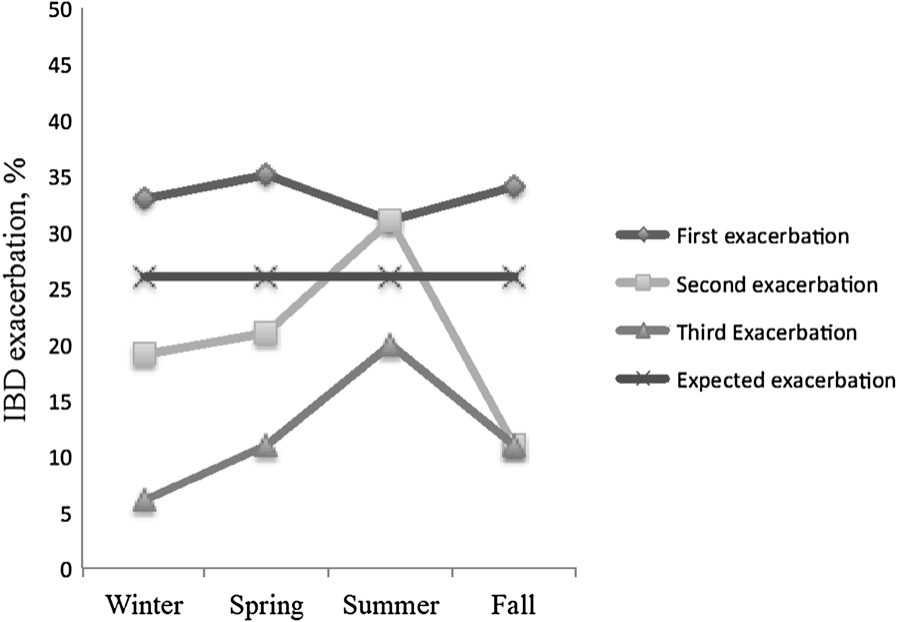
Seasonal distribution of the first three IBD exacerbations as compared to expected pattern of exacerbations expressed as percentage in a 3-D line graph. No difference was detected in seasonality between the observed and expected pattern of IBD exacerbations for the first three exacerbations experienced by patients (*p* values were 0.066, 0.250, and 0.17 respectively). *IBD* inflammatory bowel diseases

A total of 358 exacerbations were determined from the data collected on 170 patients with IBD. The median number of exacerbations was two ranging from 1 to 11. IBD exacerbations were found to be uniformly distributed throughout the year (Fig. 4) and no difference was detected in seasonality between the observed and expected pattern of IBD exacerbations for the first three exacerbations experienced by patients (*p* values were 0.066, 0.250, and 0.17 respectively; Fig. 5). Patients with either CD or UC did not appear to follow any seasonal pattern with their exacerbations, as there was no significant difference in the seasonal distribution of their first three exacerbations (Table 2).

**Table 2:** Seasonal pattern of first three exacerbations of IBD

Type of IBD	Number of exacerbations	Season					
		Winter	Spring	Summer	Fall	Total	*p*
		n (%)	n (%)	n (%)	n (%)	n (%)	
CD	First	20 (25.3 %)	12 (27.8 %)	19 (24.1 %)	18 (22.8 %)	79 (100 %)	0.695
	Second	9 (21.4 %)	12 (28.6 %)	17 (40.5 %)	4 (9.5 %)	42 (100 %)	0.103
	Third	3 (12 %)	5 (20 %)	12 (48 %)	5 (20 %)	25 (100 %)	0.138
UC	First	13 (20.3 %)	23 (35.9 %)	12 (18.8 %)	16 (25 %)	64 (100 %)	0.450
	Second	10 (25 %)	9 (22.5 %)	14 (35 %)	7 (17.5 %)	40 (100 %)	0.614
	Third	3 (13 %)	6 (26.1 %)	8 (34.8 %)	6 (26.1 %)	23 (100 %)	0.602

*IBD* inflammatory bowel diseases, *UC* ulcerative colitis, *CD* Crohn’s disease

## Discussion

It is well known that environmental factors play an important role in the pathophysiology of IBD. However, the relationship between seasonality and natural history or births of IBD patients requires further study since the results to date have been inconsistent in terms of seasonal variations in the onset and flare-up of IBD patients. Data from the present study shows that the onset of IBD symptoms tends to have a seasonal trend with the highest incidence in the fall and the lowest in the summer. However, we did not observe any association between the season and the exacerbation of IBD in the pediatric population. Moreover, there has been no specific season when children with IBD tend to be born.

The data emerging from this present study indicates that the onset of IBD symptoms occurred more frequently during the fall than during other seasons. When considering CD and UC separately, the clinical onset of these two diseases was clustered in the fall, although clustering did not reach statistical significance. Few studies concerning factors that may trigger the onset and exacerbation of IBD have been published, mostly from adult populations. Although several infectious agents likely play a role in the pathogenesis of IBD, there is no conclusive evidence of for specific microbial agents functioning as etiological factors [20–23]. Viral respiratory infections are generally more common in fall and winter, which is probably due to a variety of factors including school being in session, increased risk for exposure as people tend to stay indoors and are in closer proximity with each other, and low humidity causing dry nasal passages that are more susceptible to cold viruses. Infections with enterovirus, salmonella and campylobacter also peak in late summer and fall [24–26]. Seasonal exposure to these infectious agents may induce bursts of immune diseases, modulating the gut immune response and contributing to intestinal inflammation. Use of antibiotics and non-steroidal anti-inflammatory drugs (NSAIDs) also seems to occur more frequently during these seasons due to the seasonality of infections resulting in an alteration of intestinal microflora and intestinal mucosal damage, which can also play a role in the etiology of IBD [27–29]. As IBD is more common in the northern parts of the United States rather than the southern parts, climatic changes might also trigger the clinical onset of IBD in children [30]. At the present time the nature of the mechanisms that underlie these fluctuations remains speculative. In future studies it would be worthwhile to correlate the temporal changes of IBD with various seasonal factors.

IBD is triggered by an inappropriate immune activation in genetically predisposed individuals. Exposure to seasonally variable external factors during the maturation of the immune system is thought to be an inducing factor for IBD, with the immune response varying in different seasons. The seasonal fluctuations of immune function in different seasons may also explain the seasonal variation in symptom onset. A change in the production of various pro-inflammatory cytokines, immunoglobulins, adrenal corticosteroid hormones and melatonin, and altered Th1/Th2 ratios that influence immune function may also be responsible for triggering the clinical onset of IBD [31–39].

Our study failed to show any association with seasonality and exacerbations of IBD within the pediatric population. We also attempted to determine if the underlying disease (either CD or UC) had any bearing on the seasonality of the relapses. When we compared the two sub-groups, patients in both categories had no significant difference with regards to the seasonal distribution of their exacerbations; thus, there is likely no seasonal trend for either group. In the past seasonal variation in exacerbations of IBD was found mostly in small samples of data obtained from the records of individual hospitals or endoscopy centers. Researchers using larger datasets have been unable to confirm any clear-cut periodicity in the occurrence of the disease. However, we do not rule out the influence of environmental factors in IBD exacerbations as our data showed an association between the seasons and IBD onsets. Moreover, both the onset and exacerbations seem to be influenced by identical modulators of disease activity. We also determined the seasonal pattern in births of patients with IBD; the distribution of births showed no statistical difference when analyzed on a seasonal basis and therefore our data is consistent with that obtained from other studies [14, 19].

We acknowledge several limitations of this present study. Our study was retrospective in nature and was a single center study. The data are, therefore, likely to be biased by documentation of clinical records and regional practice patterns. We studied seasonal changes and did not consider variations that occur in individual months. There is also a possibility that a small proportion of the exacerbations may have gone undocumented, either because the symptoms were so mild that the patients did not seek treatment or because the exacerbation was treated at a different site. However, the strength of this study is that it analyzed a large number of children with IBD throughout their clinical course. We attempted to decrease the effect of the recall bias by using the calendar season and by excluding patients who claimed that their symptoms began more than 6 months prior to visiting the GI clinic. Our study was a unique attempt to address the seasonality of IBD in the pediatric population.

## Conclusions

In conclusion, our data suggests that the onset of symptoms of IBD in children tends to have a seasonal trend with the highest incidence in the fall and the lowest in the summer. However, neither exacerbations nor birth cases were influenced by any underlying seasonal variations. Seasonal factors may be responsible for the periodicity of IBD onsets and we recommend further studies to identify the role of these exposures (if any) that could influence the seasonality of IBD in children.

## Abbreviations

IBD: inflammatory bowel diseases
CD: Crohn’s disease
UC: ulcerative colitis
IC: indeterminate colitis
ICD: International Classification of Diseases
SD: standard deviation
SPSS: statistical package for social science
NSAIDs: non-steroidal anti-inflammatory drugs
